# Prevalence and clinical phenotype of the triplicated α-globin genes and its ethnic and geographical distribution in Guizhou of China

**DOI:** 10.1186/s12920-021-00944-9

**Published:** 2021-04-07

**Authors:** Xi Luo, Xiang-mei Zhang, Liu-song Wu, Jindong Chen, Yan Chen

**Affiliations:** 1 Department of Pediatrics, Affiliated Hospital of Zunyi Medical University, Zunyi, 563000 Guizhou China; 2 Department of Medical Genetics, Zunyi Medical University, Zunyi, 563000 Guizhou China; 3 Exploring Health LLC, Guangzhou, 510663 Guangdong China; 4 Department of Urology, University of Rochester Medical Center, Rochester, NY 14642 USA

**Keywords:** Population genetics, Epidemiology, Prevalence, Triplicated α-globin genes, α-globin triplication, β-thalassemia, Anemia

## Abstract

**Background:**

α-thalassemia is relatively endemic in Guizhou province of southwestern China. To predict the clinical manifestations of α-globin gene aberration for genetic counseling, we examined the prevalence of the α-globin triplication and the genotype–phenotype correlation in this subpopulation

**Methods:**

A cohort of 7644 subjects was selected from nine ethnicities covering four regions in Guizhou province of China. Peripheral blood was collected from each participant for routine blood testing and hemoglobin electrophoresis. PCR-DNA sequencing and Gap-PCR were used to identify the thalassemia gene mutations. Chi-square tests and one-way analysis of variance (ANOVA) were used to statistically analyze the data.

**Results:**

We found that the frequency of α-globin triplication in Guizhou province was 0.772% (59/7644). Genotypically, the ααα^anti4.2^/αα accounted for 0.523% (40/7644), the ααα^anti3.7^/αα for 0.235% (18/7644), and the ααα^anti3.7^/–^SEA^ for 0.013% (1/7644). The ααα^anti4.2^/αα is more prevalent than the ααα^anti3.7^/αα in Guizhou. In addition, the frequency of the HKαα/αα (that by GAP-PCR is like ααα^anti4.2^/-α^3.7^) was 0.235% (18/7644). Ethnically, the Tujia group presented the highest prevalence (2.47%) of α-globin triplication. Geographically, the highest frequency of the α-globin triplication was identified in Qiannan region (2.23%). Of the triplicated α-globin cases, 5 coinherited with heterozygote β-thalassemia and presented various clinical manifestations of anemia.

**Conclusions:**

These data will be used to update the Chinese triplicated α-globin thalassemia database and provide insights into the pathogenesis of thalassemia. These findings will be helpful for the diagnosis of thalassemia and future genetic counseling in those regions.

## Background

**Fig. 1:**
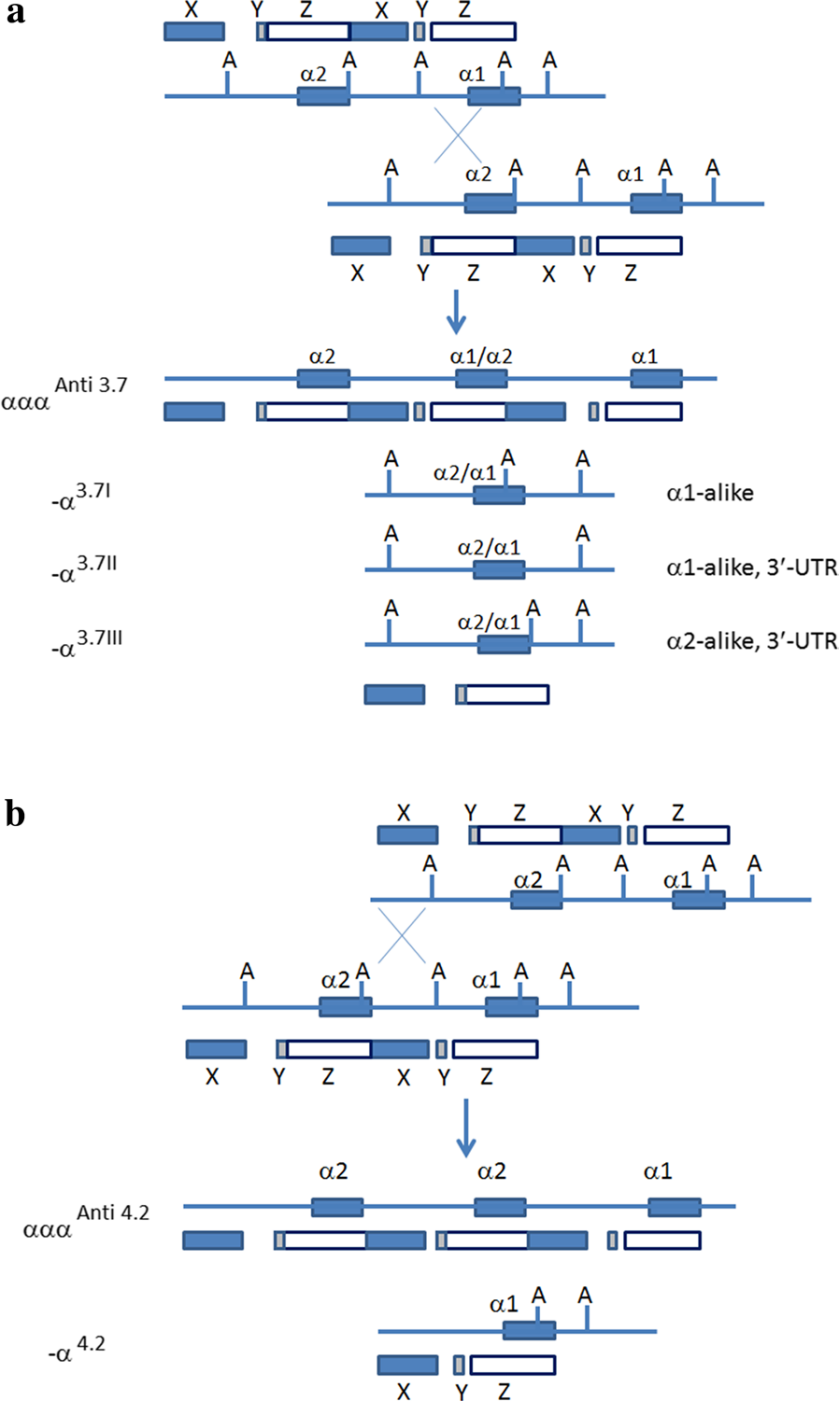
Schematic generation of the two α-triplications (ααα^anti3.7^ and ααα^anti4.2^) through homologous recombination between the duplicated α-globin genes. **a** Generation of the ααα^anti3.7^ triplication and the three subtypes of rightward deletion (-α^3.7I,II,III^) due to unequal crossing over between two misaligned Z boxes of the α1- and α2-globin genes and reciprocal events; **b** Generation of the ααα^anti4.2^ triplication and deletion of –α^4.2^ from recombination between the two misaligned X-homology boxes. *Note*: X, Y, Z, homology boxes; A, *Apa* I restriction site

Thalassemia is a hereditary hemoglobin disease caused by defects in the globin genes, including deletions and mutations [1]. Based on the gene involved, thalassemia is usually classified into α-thalassemia and β-thalassemia [2, 3]. While a deletion of one or both α-globin genes leads to α-thalassemia, the α-globin genes triplication (ααα) that caused by homologous recombination between the duplicated α-globin genes (Fig. 1), rarely causes detectable clinical symptoms because the clinical blood parameters and manifestations appear normal [4–6]. However, when in coinherited with β-globin gene mutation(s), the triplicated α-globin genes play a considerable role in pathophysiology of thalassemia by deteriorating the imbalanced α-globin chain synthesis and affecting the erythroid maturation and survival [7, 8], mild to severe thalassemia (transfusion-dependent anemia) is often observed in the affected subjects due to the imbalance of α- and β-globin chains [8–10]. Patients with severe thalassemia usually rely on lifelong blood transfusion therapy, which is a heavy healthcare burden for their families and society. There are two types of triplicated α-globin genes: ααα^anti3.7^ and ααα^anti4.2^ [11, 12]. The ααα^anti4.2^ is commonly observed in Asians while the ααα^anti3.7^ is more prevalent in Africans, Middle Eastern, and Mediterranean populations [7, 11–13]. In addition, a type of unusual rearrangement of the α-globin gene cluster, called HKαα (Hong Kong αα) allele, contains both the -α^3.7^ and ααα^anti4.2^ crossover junctions [14, 15]. But the HKαα allele does not really contain three copies of α-globin gene. Thus, HKαα is not an α-triplication allele.

Guizhou province, located in southwestern China, is one of the regions with the highest rate of α-thalassemia in Asia [16]. The population consists of several ethnic groups including many minorities such as Yao, Miao, Buyi, Dong, Tujia, Zhuang, Shui and Gelao. Although thalassemia patients with β-globin gene defects and triplicated α-globin genes had been reported worldwide [9, 17, 18], the frequency of triplicated α-globin genes in this population has never been investigated. Thus, we conducted an epidemiological study to elucidate the frequency and clinical features of triplicated α-globin genes in this population and region.

## Methods

### Subjects

**Fig. 2:**
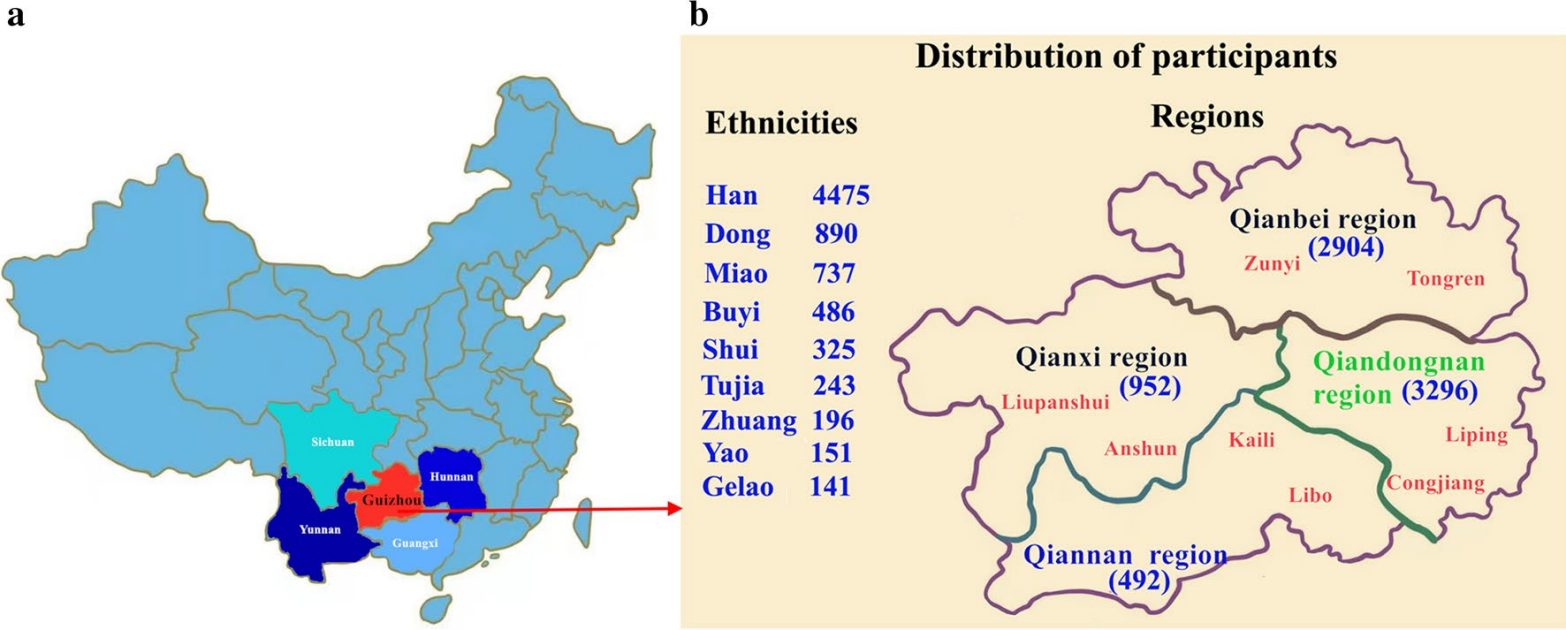
Map of the sampling location. **a** Guizhou province is located in southwestern China; **b** Blood samples were randomly collected from 8 counties/cities in four regions of Guizhou province, covering nine ethnic groups. Note: the maps were drawn by tracing existing maps through software Procreate (version 5.0.5, Savage Interactive Pty Ltd.), and owned by authors in this study

Guizhou province contains four regions. Two representative counties/cities from each region were taken for study. Inclusion criteria: subjects whose residence in these regions exceeded 3 years at the time of recruitment, regardless of age, sex, and ethnicity. In total, 7866 participants were recruited by simple random sampling method from 8 counties/cities (Congjiang, Liping, Tongren, Libo, Liupanshui, Kaili, Zunyi, and Anshun) in four regions (Qiannan, Qiandongnan, Qianbei and Qianxi) of Guizhou province in China from February 2014 to June 2016. Eventually, blood samples and health information were collected from 7644 qualified people for investigation based on the inclusion criteria (Fig. 2).

### Blood analysis

Approximately 5 ml peripheral blood was collected from each participant for routine blood testing (Sysmex hematology analyzer, K-1000, Sysmex Corporation, Kobe, Japan) and hemoglobin electrophoresis (Bio-Rad Laboratories, Hercules, CA, USA).

### DNA sequencing and genotyping

Approximately 3 ml of peripheral blood was collected from each subject. DNA extraction was conducted by using the Magen nucleic acid extraction kit (Magen, Guangzhou, China). Four pairs of globin gene specific PCR primers (HBA1, HBA2, HBB-1, and HBB-2) were designed and synthesized by the Beijing Genome Institute (BGI)-Shenzhen. For identification of HKαα, next-generation sequencing plus Gap-PCR were adopted. All the globin gene-specific PCR primers were owned and patented by the BGI-Shenzhen, and unpublicized. PCR was carried out in a volume of 25 μl with an amplification reaction system containing 1 pair of tag primers, 50–200 ng DNA, and 2 × Gold Star Taq Master Mix (Kangwei century). The PCR amplification was performed using the ABI9700 (Perkin-Elmer Applied Biosystems Inc., Foster City, CA). The PCR conditions: 95 °C 10 min; 95 °C 30 s, 60 °C 30 s, 72 °C 50 s, 35 cycles; 72 °C 5 min, 15 °C hold; PCR conditions for copy number variations: 95 °C 10 min; 95 °C 30 s, 60 °C 60 s, 24 cycle; 15 °C hold. Whole genome DNA sequencing was performed to detect globin gene defects by the Beijing Genome Institute (BGI)-Shenzhen through next-generation sequencing technology [19]. Gap-PCR was used to detect some α deletion genotypes, as described previously [15, 19]. Information from subjects with triplicated α-globin genes combined with β-thalassemia clinical manifestations were collected for further analysis. The nomenclature and description of the α-globin gene variants identified followed the HGVS guidelines (http://www.HGVS.org/varnomen).

### Statistical analysis

Continuous variables are summarized by descriptive statistics, including the mean and range or standard deviation. Categorical variables are presented as number and percentage, and the comparisons of frequencies and mean were completed by using the Chi-square test and one-way analysis of variance (ANOVA). A statistically significant difference was defined as a p < 0.05. Statistical analyses were performed with SPSS 17.0 (SPSS Inc., Chicago, IL, USA).

## Results

### Population samples

Of the 7644 qualified participants, 3817 were males and 3827 were females, the participants’ ages ranged from 5 to 68 (average age 25 ± 3.4) years, 1027 (13.44%) were minors, and 6617 (86.56%) were adults. The geographical residence pattern of the subjects was as follows: Qianbei (Zunyi, Tongren) region, 2904 (37.99%); Qiannan (Kaili, libo), 491 (6.42%); Qianxi (Liupanshui, Anshun), 952 (12.45%); and Qiandongnan (Congjiang, Liping), 3297 (43.13%). There were nine ethnicities inhabiting those regions: Han, 4475 (58.5%); Dong, 890 (11.6%); Miao, 737 (9.6%); Buyi, 486 (6.4%); Shui, 325 (4.3%); Tujia, 243 (3.2%); Yao, 151 (2%); Gelao, 141 (1.8%); and Zhuang 196 (2.6%).

### Prevalence of triplicated α-globin genes and the HKαα allele

As listed in Table 1, among the 7644 subjects examined, 59 carried heterozygous triplicated α-globin genes. The prevalence of the triplicated α-globin genes was 0.772% or 772/100,000 in this subpopulation. The ααα^anti4.2^/αα accounted for 0.523% (40/7644), the ααα^anti3.7^/αα for 0.235% (18/7644), and 1 subject carried a ααα^anti3.7^ allele and a –^SEA^ allele (genotype: ααα^anti3.7^/–^SEA^), accounting for 0.013% (1/7644). Thus, the ααα^anti4.2^/αα is more prevalent than the ααα^anti3.7^/αα in Guizhou province (*p* < 0.05). In addition, we identified 18 cases of the HKαα/αα carriers, accounting for 0.235% (18/7644).

**Table 1:** Prevalence of each type of the α-globin gene triplication and HKαα allele in 7644 subjects

Genotype	Triplicated α-globin gene carriers (%)		Total (%)
	Male	Female	
ααα^anti 4.2^/αα	22 (0.288)	18 (0.235)	40 (0.523)
ααα^anti 3.7^/αα	10 (0.13)	8 (0.105)	18 (0.235)
HKαα/αα	5 (0.065)	13 (0.17)	18 (0.235)
ααα^anti3.7^/–^SEA^	1 (0.013)		1 (0.013)

### The ethnic distribution of the triplicated α-globin genes and the HKαα allele

As listed in Table 2, there are 9 ethnic groups inhabiting these regions. Except for the Han ethnic group, the other ethnic groups are minorities in China. The ethnicity of the participants was determined by questioning. In this investigation, the highest frequency of the ααα^anti4.2^/αα was identified in the Tujia ethnic group (1.65%), followed by the Han, Dong, Shui, Buyi, and Miao. The frequency of the ααα^anti4.2^/αα in the Tujia group was significantly higher than in the Miao (*p* = 0.015), Dong (*p* = 0.022), and Buyi (*p* = 0.045). For the ααα^anti 3.7^/αα genotype, the highest rate was observed in the Tujia group as well, followed by the Dong, Han, Buyi, and Miao. No ααα^anti 3.7^/αα carrier was observed in the Shui group. No triplicated α-globin genes were detected in the Zhuang, Yao, and Gelao groups.

**Table 2:** The ethnic distribution of the triplicated α-globin genes and HKαα allele in Southwestern Guizhou

Ethnicity (number)	ααα^anti 4.2^/αα (%)	ααα^anti 3.7^/αα (%)	HKαα/αα (%)	ααα^anti3.7^/–^SEA^ (%)	Total
Han (n = 4475)	31 (0.69)	11 (0.25)	12 (0.27)	1 (0.022)	55 (1.23)
Dong (n = 890)	2 (0.22)	3 (0.34)	3 (0. 34)	0	8 (0.90)
Miao (n = 737)	1 (0.14)	1 (0.14)	1 (0.14)	0	3 (0.42)
Buyi (n = 486)	1 (0.21)	1 (0.21)	1 (0.21)	0	3 (0.63)
Shui (n = 325)	1 (0.31)	0	1 (0.31)	0	2 (0.62)
Tujia (n = 243)	4 (1.65)	2 (0.82)	0	0	6 (2.47)
Zhuang (n = 196)	0	0	0	0	0
Yao (n = 151)	0	0	0	0	0
Gelao (n = 141)	0	0	0	0	0

### The geographical distribution of the triplicated α-globin genes and the HKαα allele

As listed in Table 3, the highest prevalence of the α-globin gene triplication was observed in Qiannan region (2.23%), followed by Qiandongnan, Qianbei, and Qianxi. The frequency in Qiannan was significantly higher than in any other regions including Qiandongnan (*p* = 0.03), Qianbei (*p* = 0.001), and Qianxi (*p* = 0.0045). There was no significant difference observed between the other regions. While the prevalence of the ααα^anti 4.2^/αα genotype was significantly higher in Qiannan region than in other regions, the ααα^anti3.7^/αα was commonly found in Qiannan and Qiandongnan regions. However, although the frequency of the ααα^anti3.7^/αα was higher in Qiandongnan, there was no statistically significant distribution difference between those regions (*p* > 0.05). In addition, the HKαα/αα was mainly distributed in Qiandongnan, but its distribution had no statistically significant difference between those regions (*p* > 0.05). The ααα^anti3.7^/– ^SEA^ was quite rare in those regions, and only one case was detected in Qiandongnan.

**Table 3:** The geographical distribution of the triplicated α-globin genes and HKαα allele in Southwestern Guizhou

Regions (number)	ααα^anti 4.2^/αα (%)	ααα^anti 3.7^/αα (%)	HKαα/αα (%)	ααα^anti3.7^/–^SEA^ (%)	Total
Qiandongnan (n = 3296)	16 (0.49)	11 (0.33)	16 (0.49)	1 (0.03)	44 (1.34)
Qianbei (n = 2904)	12 (0.41)	6 (0.21)	0	0	18 (0.62)
Qiannan (n = 492)	9 (1.83)	1 (0.2)	1 (0.2)	0	11 (2.23)
Qianxi (n = 952)	3 (0.32)	0	1 (0.11)	0	4 (0.43)

%, percentage, the number of a specific genotype carriers divided by the participants examined in a specific region

### Genotype–phenotype associations of α-globin gene rearrangements

The frequency of thalassemia-gene carriers in Guizhou is 11.03%, with 7.41% of α-thalassemia-gene frequency and 3.23% of β-thalassemia-gene frequency (unpublished data). Therefore, the frequency of α-thalassemia is higher than β-thalassemia in Guizhou province, China. While deletion of α-globin genes causes α-thalassemia, the triplicated α-globin genes alone rarely cause obvious clinical symptoms. All 59 carriers of the triplicated α-globin genes including the ααα^anti3.7^/–^SEA^ case, and 18 cases of the HKαα/αα did not presented any clinical manifestations such as anemia at the time of examination. The blood parameters including the red blood cells (RBC), hemoglobin (HGB), mean corpuscular volume (MCV), mean corpuscular hemoglobin (MCH), HbA, HbA2, and HbF were measured and statistically analyzed through ANOVA. No significant parameter difference was identified among the three different genotype groups (*p* > 0.05) (Tables 4, 5). All of the hematological parameters appeared within the normal range. However, when triplicated α-globin genes coinherit with β-globin gene mutation(s), the affected subjects present various clinical manifestations from no symptoms to severe anemia. In this study, we identified 5 subjects who were cocarriers of the β-globin gene alterations and the α-globin gene triplication. All 5 affected subjects were of Han ethnicity. Mut-01 was a boy who suffered from severe anemia when he was 7 years old and was treated with regular blood transfusions at a local hospital since he was diagnosed with β-thalassemia. Our DNA sequencing demonstrated that he was a carrier of codons 41/42 (-TTCT) beta^0^ (HBB: c.126_129delCTTT) and αα/ααα^anti4.2^ (Table 6). Mut-02 and Mut-03 did not present with severe symptoms but exhibited visible paleness. No abnormalities were observed in their hearts, lungs, and nervous systems. The blood tests indicated that all three boys suffered from moderate hypochromic microcytic anemia without detectable iron deficiency or other related abnormalities. Mut-04 had the identical β-globin mutation as Mut-01, and his disease manifestation was similar to that of Mut-01. Mut-04′s father was also a β-thalassemia sufferer (HGB, 83 g/L; MCV, 62.2 fL; MCH, 19.6). DNA sequencing indicated that they were all carriers of the β-globin gene alterations and the α-globin gene triplication (Table 6). Mut-05 was an adult woman. She had no detectable symptoms at the time of examination, although she carried a codon 17 (AAG > TAG) beta^0^ (HBB:c.52A > T) and the αα/ααα^anti4.2^.

**Table 4:** The blood parameters of the α-globin gene triplication and HKαα groups

Genotypes	Number	RBC (× 10^12^/L)	HGB (g/L)	MCV (fL)	MCH (pg)
ααα^anti3.7^/αα	16	5 ± 0.27	138 ± 9.8	93 ± 7.7	31 ± 2.7
HKαα/αα	18	4.6 ± 0.31	125.9 ± 12.7	88.6 ± 8.37	28.51 ± 2.28
ααα^anti4.2^/αα	37	5 ± 0.46	133.9 ± 13	93 ± 9.5	30 ± 1.7
ααα^anti3.7^/–^SEA^	1	4.9	135	86.5	29.8

RBC, red blood cells (normal range: 4–5 × 10^12^/L); HGB, hemoglobin (normal range: male, 120–170 g/L; female, 110–160 g/L); MCV, mean corpuscular volume (normal range: 80–100 fL); MCH, mean corpuscular hemoglobin (normal range: 27–32 pg)

**Table 5:** The hemoglobin electrophoresis data of the α-globin gene triplication and HKαα groups

Genotypes	Number	HbA (%)	HbA2 (%)	HbF (%)
ααα^anti3.7^/αα	16	97 ± 0.21	3 ± 0.21	–
HKαα/αα	18	96.9 ± 1.0	2.89 ± 0.37	0.7 ± 0.2
ααα^anti4.2^/αα	37	97 ± 0.44	3 ± 0.31	0.8 ± 0.2

–, no data

**Table 6:** Hematological parameters of the 5 heterozygous cocarriers of the β-globin gene mutation and the α-globin gene triplication

Subject ID	Mut-01	Mut-02	Mut-03	Mut-04	Mut-05
β-gene defects	Cds 41/42 (-TTCT) beta^0^	Cds 71/72 (+ A) beta^0^	IV-II-654 (C > T) beta^+^	Cds 41/42 (-TTCT) beta^0^	Cd17 (AAG > TAG) beta^0^
α genotypes	αα/ααα^4.2^	αα/ααα^3.7^	αα/ααα^3.7^	αα/ααα^4.2^	αα/ααα^4.2^
Anemia	severe	mild	mild	mild	no
Sex	M	M	M	M	F
Age (years)	14	5	7	7	19
HGB (g/L)	59	80	85	78	134
MCV (fL)	58	68	73	59.6	97.5
MCH	17	25	24	18.5	30.9
HbA	–	97.7	90	91.9	97.5
HbA2	–	4.3	3.7	5.8	2.5
HbF	–	–	–	2.3	–
Hepato-splenomegaly	Splenomegaly	–	–	Splenomegaly	–
Transfusion	Y	N	N	N	N
Treatment and prognosis	b	a	a	c	a

Cd, codon; M, male; F, female; Y, yes; N, no; a, no treatment and occasional follow-up; b, transfusion treatment (HGB < 80 g/L, transfused with washed red blood cells) and regular follow-up; c, transfusion treatment (HGB < 80 g/L, transfused with washed red blood cells) and occasional follow-up

## Discussion

To date, at least two genotypes of α-globin triplication have been described: ααα^anti4.2^/αα, ααα^anti3.7^/αα. Although the HKαα shows by GAP-PCR the positivity for ααα^anti4.2^ and -α^3.7^, it is not considered as α-globin triplication due to no real extra copy of the α-globin gene existed in the HKαα allele. Observations have indicated that α-globin triplication alone does not cause detectable clinical manifestations [20]. However, when an α-globin triplication coinherits with β-globin gene mutation(s), the combined defects usually lead to the emergence of variable clinical phenotype including asymptomatic presentation, significant anemia, ineffectual erythropoiesis, and mild to severe clinical symptoms. Thus, it is essential to determine the prevalence of the triplicated α-globin genes because the α-globin triplication usually exacerbates β-thalassemia when it is coinherited with β-globin defects because the extra copy of the α-globin chain leads to an unbalanced ratio between the α- and β-globin chains if associated with β-thalassemia. In this study, we randomly selected a cohort of 7644 subjects in four regions of Guizhou province, China. These participants’ ethnicities included Han and eight ethnic minorities. Our study demonstrated that the population prevalence of the α-globin triplication in Guizhou province was 0.772%. This figure was slightly lower than that identified in Guangdong (1.2%), a province in southeastern China [17], and in the Dutch population (approximately 1.2%) [7]. In addition, the ratio of ααα^anti3.7^ and ααα^anti4.2^ was different between the two subpopulations. In our study, the ratio of ααα^anti3.7^/ααα^anti4.2^ was 0.47 (0.248% ααα^anti3.7^/0.523% ααα^anti4.2^) in Guizhou province, while it was 3.0 (0.9% ααα^anti3.7^/0.3% ααα^anti4.2^) in Guangdong province. This finding suggests that the ααα^anti4.2^ triplication is rather common in Guizhou, while the ααα^anti3.7^ is prevalent in Guangdong region.

Ethnically, the Tujia group presented the highest prevalence (2.47%) of the α-globin triplication. In particular, the prevalence of ααα^anti4.2^ in Tujia was significantly higher (1.65%) than in any other ethnic group. This is the first report that Tujia have a higher frequency of α-globin triplication. Whether this higher rate of α-globin triplication is caused by a founder effect or not requires further investigation. Although previous studies have reported that the frequencies of α-globin defects in the Zhuang and Yao ethnic groups were significantly higher than that in the Han ethnic group in Guangxi, another province in southwestern China [21], we did not observe any carrier of α-globin triplication in Zhuang and Yao minorities, probably due to the small size of the ethnic groups or that they were not affected by the α-globin triplication in those regions. The high frequency of α-globin triplication identified in those two ethnic groups in Guangxi province might be caused by a founder effect initiated by genetic drift and particular lifestyles, inhabitation density, and endogamous marriage.

Geographically, the highest frequency of the α-globin triplication was identified in Qiannan region (2.23%). The frequency difference of the α-globin triplication between Qiannan and any other region was statistically significant. Moreover, the frequency of ααα^anti4.2^ in Qiannan was also higher than in other regions. The other type of α-globin triplication, ααα^anti3.7^/–^SEA^, and the non-α-triplication HKαα/αα had no significant geographical differences. In addition, we did not identify any anti-HKαα allele, although we found that the frequency of HKαα allele was 0.235%, significantly higher than previously reported. To exclude the presence of HKαα in case of positivity for the -α^3.7^ and ααα^anti4.2^, next-generation and Gap-PCR are needed to be simultaneously performed. Interestingly, while the HKαα is hardly observed in other regions, it was relatively common in Qiandongnan and its frequency is comparable to that of the ααα^anti 4.2^ triplication there (Table 3). The HKαα was first described by Wang [14]; then, Shang et al. first reported that the population prevalence of HKαα was 0.07% and that of anti-HKαα was 0.02% in Guangxi province of China [22]. Afterwards, the population prevalence of HKαα was determined to be 0.07% and that of anti-HKαα to be 0.003% in Guangdong province [23]. The differences between our findings and the previously reported data could be due to geographical pattern differences and population diversity [24].

In our study, the hematological parameters and hemoglobin electrophoresis data of the HKαα, ααα^anti4.2^, and ααα^anti3.7^ carriers were all within the normal range, which is consistent with previous reports [14, 22]. Therefore, carriers of the HKαα, ααα^anti4.2^, and ααα^anti3.7^ will not present clinical manifestations such as anemia. Of note, although the HKαα carriers presented a normal range of hematological parameters, their RBC, hemoglobin, MCH, and MCV were all slightly lower than the ααα^anti4.2^ and ααα^anti3.7^ carriers, implying that the particular cluster structure that could reduce the α-globin gene expression.

As mentioned above, the triplicated α-globin genes alone barely lead to detectable clinical phenotypes. In this study, of the 59 cases of α-globin genes triplication, 5 cases coinherited with β-globin gene mutation(s) while the other 54 subjects did not present any clinical symptoms. Although many reports have stated that α-globin triplication can exacerbate the symptoms of β-thalassemia, the issue is still controversial because the expected worsened anemia has not occurred in all cases [7, 18]. In our subjects, the first four carriers presented with β-thalassemia from mild to severe. In the case of Mut-05, the α-globin triplication combined with the β-globin gene mutation (CD17 (AAG > TAG)) surprisingly failed to cause thalassemia; the reason merits further investigation. In addition, 18 cases of the HKαα/αα, and 1 case of the ααα^anti3.7^/–^SEA^ did not presented any clinical manifestations such as anemia at the time of examination, which is consistent with previous reports [22, 23].

Currently, Gap-PCR and PCR combined with RDB (reverse dot blot) methods are commonly used to detect α-globin gene deletions and the β-globin gene defects, but they usually miss the triplicated α-globin genes. In this study, whole genome NGS combined with Gap-PCR was adopted to screen for all types of α-globin and β-globin gene alterations, including α-globin gene deletion, triplication, splicing mutations, which would be expected to increase the detection sensitivity and improve the diagnosis of β-thalassemia.

## Conclusions

This epidemiological study has identified the current α-triplication genotypes and their prevalence and distribution in Guizhou province, which will be used to update the triplicated α-globin thalassemia database, provide insights into the pathogenesis of thalassemia and shed light on the diagnosis of thalassemia in southwestern China.

## Data Availability

The authors declare that the data supporting the results of this study are provided in this paper. All original data used in this study have been deposited in NCBI Trace Archive or NCBI Sequence Read Archive (SRA). Accession number for these SRA data is PRJNA703755 (https://www.ncbi.nlm.nih.gov/bioproject/PRJNA703755/, and download link: https://www.ncbi.nlm.nih.gov/Traces/study/?acc=PRJNA703755).

## Abbreviations

RBC: Red blood cells
HGB: Hemoglobin
MCV: Mean corpuscular volume
MCH: Mean corpuscular hemoglobin
RDB: Reverse dot blot
ANOVA: One-way analysis of variance
PCR: Polymerase chain reaction

## References

[1] Higgs DR, Engel JD, Stamatoyannopoulos G (2012). Thalassaemia. Lancet.

[2] Muncie HL, Campbell J (2009). Alpha and beta thalassemia. Am Fam Phys.

[3] Su Q, Chen S, Wu L, Tian R, Yang X, Huang X, Chen Y, Peng Z, Chen J (2019). Severe thalassemia caused by Hb Zunyi [beta147(HC3)Stop–>Gln; HBB: c.442T>C)] on the beta-globin gene. Hemoglobin.

[4] Propper RD (1980). Hemolytic anemia: thalassemia syndromes. Pediatr Ann.

[5] Weatherall DJ (1980). The thalassemia syndromes. Texas Rep Biol Med.

[6] Vichinsky E (2012). Advances in the treatment of alpha-thalassemia. Blood Rev.

[7] Giordano PC, Bakker-Verwij M, Harteveld CL (2009). Frequency of alpha-globin gene triplications and their interaction with beta-thalassemia mutations. Hemoglobin.

[8] Abedini SS, Forouzesh Pour F, Karimi K, Ghaderi Z, Farashi S, Tavakoli Koudehi A, Javadi Pirouz H, Mobini Nejad SB, Azarkeivan A, Najmabadi H (2018). Frequency of alpha-globin gene triplications and coinheritance with beta-globin gene mutations in the Iranian population. Hemoglobin.

[9] Farashi S, Bayat N, Faramarzi Garous N, Ashki M, Montajabi Niat M, Vakili S, Imanian H, Zeinali S, Najmabadi H, Azarkeivan A (2015). Interaction of an alpha-globin gene triplication with beta-globin gene mutations in Iranian patients with beta-thalassemia intermedia. Hemoglobin.

[10] Yus Cebrian F, Recasens Flores Mdel V, Izquierdo Alvarez S, Parra Salinas I, Rodriguez-Vigil Iturrate C (2016). Combination of a triple alpha-globin gene with beta-thalassemia in a gypsy family: importance of the genetic testing in the diagnosis and search for a donor for bone marrow transplantation for one of their children. BMC Res Notes.

[11] Lie-Injo LE, Herrera AR, Kan YW (1981). Two types of triplicated alpha-globin loci in humans. Nucleic Acids Res.

[12] Trent RJ, Higgs DR, Clegg JB, Weatherall DJ (1981). A new triplicated alpha-globin gene arrangement in man. Br J Haematol.

[13] Camaschella C, Kattamis AC, Petroni D, Roetto A, Sivera P, Sbaiz L, Cohen A, Ohene-Frempong K, Trifillis P, Surrey S (1997). Different hematological phenotypes caused by the interaction of triplicated alpha-globin genes and heterozygous beta-thalassemia. Am J Hematol.

[14] Wang W, Chan AY, Chan LC, Ma ES, Chong SS (2005). Unusual rearrangement of the alpha-globin gene cluster containing both the -alpha3.7 and alphaalphaalphaanti-4.2 crossover junctions: clinical diagnostic implications and possible mechanisms. Clin Chem.

[15] Zhang M, Huang H, Chen M, Chen L, Wang Y, Lin N, Lin Y, Xu L (2019). Frequencies and hematological manifestations of the HKalphaalpha allele in southern Chinese population. Int J Clin Exp Pathol.

[16] Yu F, Zhong C, Zhou Q, Yang Y, Li W, Liu B, Pan S, Tang K, Fang R, Jin W (2010). Genetic analysis of beta-thalassemia mutations in the minority populations of Guizhou province. Chin J Med Genet.

[17] Xie XM, Wu MY, Li DZ (2015). Evidence of selection for the alpha-globin gene deletions and triplications in a southern Chinese population. Hemoglobin.

[18] Mehta PR, Upadhye DS, Sawant PM, Gorivale MS, Nadkarni AH, Shanmukhaiah C, Ghosh K, Colah RB (2015). Diverse phenotypes and transfusion requirements due to interaction of beta-thalassemias with triplicated alpha-globin genes. Ann Hematol.

[19] Shang X, Peng Z, Ye Y, Zhang X, Chen Y, Zhu B, Cai W, Chen S, Cai R (2017). Rapid targeted next-generation sequencing platform for molecular screening and clinical genotyping in subjects with hemoglobinopathies. EBioMedicine.

[20] Goossens M, Dozy AM, Embury SH, Zachariades Z, Hadjiminas MG, Stamatoyannopoulos G, Kan YW (1980). Triplicated alpha-globin loci in humans. Proc Natl Acad Sci U S A.

[21] Xiong F, Sun M, Zhang X, Cai R, Zhou Y, Lou J, Zeng L, Sun Q, Xiao Q, Shang X (2010). Molecular epidemiological survey of haemoglobinopathies in the Guangxi Zhuang Autonomous Region of southern China. Clin Genet.

[22] Shang X, Li Q, Cai R, Huang J, Wei X, Xu X (2013). Molecular characterization and clinical presentation of HKalphaalpha and anti-HKalphaalpha alleles in southern Chinese subjects. Clin Genet.

[23] Wu MY, Li J, Li SC, Li Y, Li DZ (2015). Frequencies of HKalphaalpha and anti-HKalphaalpha Alleles in Chinese carriers of silent deletional alpha-thalassemia. Hemoglobin.

[24] Jin L, Su B (2000). Natives or immigrants: modern human origin in east Asia. Nat Rev Genet.

